# Machine learning enables long time scale molecular photodynamics simulations†
†Electronic supplementary information (ESI) available. See DOI: 10.1039/c9sc01742a


**DOI:** 10.1039/c9sc01742a

**Published:** 2019-08-05

**Authors:** Julia Westermayr, Michael Gastegger, Maximilian F. S. J. Menger, Sebastian Mai, Leticia González, Philipp Marquetand

**Affiliations:** a Institute of Theoretical Chemistry , Faculty of Chemistry , University of Vienna , 1090 Vienna , Austria . Email: philipp.marquetand@univie.ac.at; b Machine Learning Group , Technical University of Berlin , 10587 Berlin , Germany; c Dipartimento di Chimica e Chimica Industriale , University of Pisa , Via G. Moruzzi 13 , 56124 Pisa , Italy

## Abstract

Photo-induced processes are fundamental in nature but accurate simulations of their dynamics are seriously limited by the cost of the underlying quantum chemical calculations, hampering their application for long time scales. Here we introduce a method based on machine learning to overcome this bottleneck and enable accurate photodynamics on nanosecond time scales, which are otherwise out of reach with contemporary approaches. Instead of expensive quantum chemistry during molecular dynamics simulations, we use deep neural networks to learn the relationship between a molecular geometry and its high-dimensional electronic properties. As an example, the time evolution of the methylenimmonium cation for one nanosecond is used to demonstrate that machine learning algorithms can outperform standard excited-state molecular dynamics approaches in their computational efficiency while delivering the same accuracy.

## Abstract 2

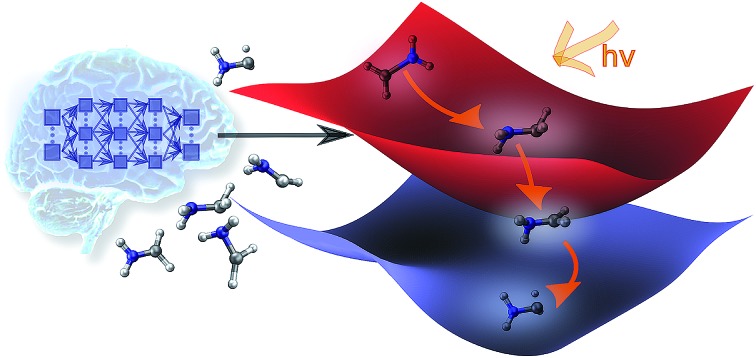
Machine learning enables excited-state molecular dynamics simulations including nonadiabatic couplings on nanosecond time scales.

## 1 Introduction

Machine learning (ML) is revolutionizing the most diverse domains, like image recognition,^1^ playing board games,^2^ or social integration of refugees.^3^ Also in chemistry, an increasing range of applications is being tackled with ML, for example, the design and discovery of new molecules and materials.^4^–^6^ In the present study, we show how ML enables efficient photodynamics simulations. Photodynamics is the study of photo-induced processes that occur after a molecule is exposed to light. Photosynthesis and DNA photodamage leading to skin cancer are only two examples of phenomena that involve molecules interacting with light.^7^–^11^ The simulation of such processes has been key to learning structure–dynamics–function relationships that can be used to guide the design of photonic materials, such as photosensitive drugs,^12^ photocatalysts^4^ and photovoltaics.^13^,^14^

**Fig. 1:**
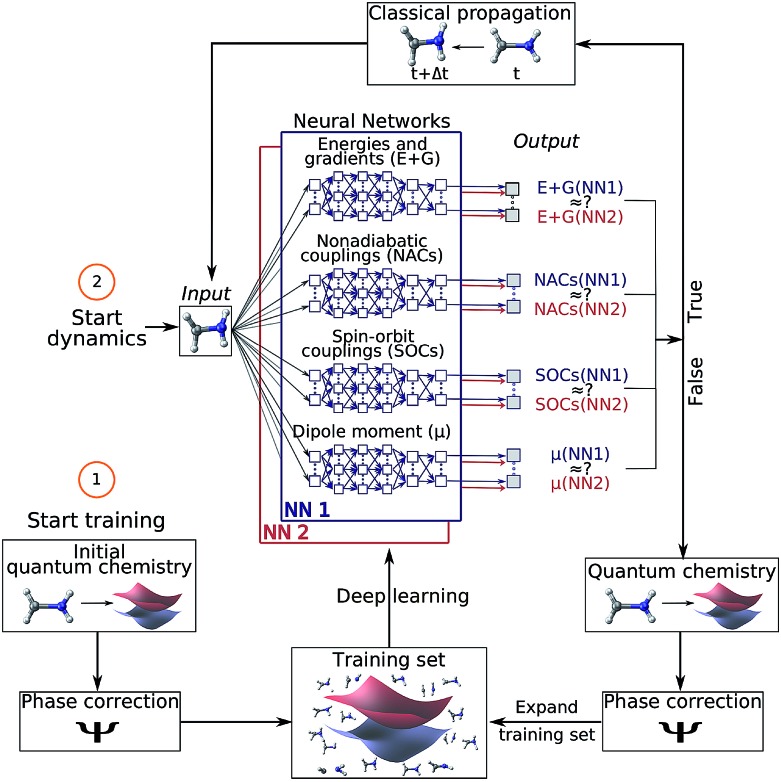
Schematic workflow of surface hopping molecular dynamics with deep NNs: the scheme starts from a set of initial quantum chemical calculations, which are pre-processed using a phase-correction algorithm and constitute an initial training set. Using this set, two deep NNs (NN1 and NN2) are trained and replace the quantum chemical calculations of energies (*E*) and gradients (*G*), nonadiabatic couplings (NACs), spin–orbit couplings (SOCs) and dipole moments (*μ*). The dynamics calculation starts with an input geometry, for which the two NNs provide all electronic quantities. If the outcomes of both NNs are sufficiently similar, the configurational space around this input geometry is adequately represented by the training set and the electronic quantities are used for a propagation time step. If not, the nuclear configuration is recomputed with quantum chemistry, phase corrected and included in the training set – a process referred to as adaptive sampling. The NNs are then re-trained and a new dynamics cycle is started.

Computer simulations of photodynamics typically rely on molecular dynamics simulations of coupled nuclei and electrons. These simulations require the computation of high-dimensional potential energy surfaces (PESs), *i.e.*, the electronic energy levels of the molecule for all possible molecular configurations, using quantum chemistry. The calculation of these PESs is usually the most expensive part of the dynamics simulations^15^ and therefore, different approximations are necessary and ubiquitous. For the electronic ground state, the time-consuming quantum chemical calculations are often replaced with force fields^16^ but no standard force fields are available to describe electronically excited states. Another drawback of most conventional force fields is their inability to describe the breaking and formation of chemical bonds. Recently, increasing effort has been devoted to ML potentials,^17^,^18^ where an accurate representation of the ground state PES including bond breaking^19^ and formation is promised.^16^,^20^–^32^ Similarly, modified Shepard interpolation is used to construct PESs in low-dimensional systems and adapt them in out-of-confidence regions.^33^,^34^ However, the problem of obtaining accurate full-dimensional PESs for excited states in order to simulate long time photodynamics has not been solved yet. A few studies have focused on the prediction of excited state dynamics as well as on excited-state properties such as spectral densities with ML.^35^–^44^ The breakdown of the Born–Oppenheimer approximation, leading to critical regions in the coupled excited state PESs,^45^ poses yet another obstacle to quantum chemistry (QC) and consequently also ML.^39^–^41^ Among those critical regions are conical intersections (or state crossings), where two PESs get into close proximity. The underlying elements that become important in such areas are nonadiabatic couplings (or spin–orbit couplings). They induce non-radiative transitions between two electronic states of the same (or different) spin-multiplicities involving ultrafast rearrangements of both nuclei and electrons. These challenges led to the need for intermittent quantum chemistry calculations^39^,^40^ or omittance of couplings between different PESs^41^ in ML driven photodynamics. Hence long time photodynamics are still lacking and the possibility to additionally represent the aforementioned nonadiabatic derivative couplings between PESs fundamental to model photodynamics has not been demonstrated yet. Here we overcome all these different bottlenecks using deep neural networks (NNs) and achieve the simulation of photodynamics for long time scales. We expand on the idea of using ML to obtain potentials for electronic excited states, as well as arbitrary couplings within a framework that combines ML with trajectory surface hopping molecular dynamics (Fig. 1). Our ML approach is fully capable of describing all necessary properties for executing nonadiabatic excited-state molecular dynamics on the order of nanoseconds. These properties include electronic energies, gradients, spin–orbit couplings, nonadiabatic couplings, and dipole moments of molecules. Additionally, the underlying potentials and couplings can be used to optimize critical points of the configurational space, such as potential minima or crossing points, which are important for interpreting photochemical mechanisms.

## 2 Theoretical background

Nonadiabatic excited-state molecular dynamics simulations are carried out using the Surface Hopping including ARbitrary Couplings (SHARC) method,^46^ which is an extension of the fewest switches surface hopping method of Tully.^47^ Within surface hopping, the nuclei are propagated according to the classical equations of motion and the electrons are treated quantum mechanically *via* interfaces to external electronic structure program packages. The electronic structure calculations are carried out on-the-fly at the nuclear geometries visited by the classical trajectories. The probability of a molecular system occupying a specific electronic state and population transfer between the different electronic states – in the form of stochastic, instantaneous hops from one electronic state to another – are dependent on the couplings between them.

### 2.1 Surface hopping molecular dynamics with deep NNs

For surface hopping simulations with NNs, the idea of retrieving electronic properties from an external source stays the same, but instead of a quantum chemical calculation, NNs are used to predict energies, gradients, couplings and dipole moments. The relationships between the nuclear coordinates and the corresponding electronic properties are learned from a training set, in which each data point is one set of nuclear coordinates and its associated set of quantities computed with a reference method. In order to make the procedure usable, the processes for generating NN potentials and their use in photodynamics simulations have been automated in a development version of the program suite SHARC.^15^,^46^,^48^

### 2.2 Training set generation and adaptive sampling for excited states

The combination of quantum chemistry with ML requires a cost-effective generation of a training set that, while it samples the conformational space of a molecular system comprehensively, is small enough to keep demanding quantum chemical reference calculations feasible.^27^ With this in mind, we employ an initial training set based on normal mode scans and then switch to an adaptive sampling scheme^21^,^24^,^49^–^51^ that automatically identifies untrustworthy regions not covered by the initial training set. The adaptive sampling procedure employs excited-state dynamics simulations using two or more NNs that are independently trained from the same training set. At every time step, the root mean squared error (RMSE) between the predictions of the different NNs of each property is compared to a predefined threshold. A separate threshold is set for each property (initially based on the validation error of the respective NN). Whenever any one of the thresholds is exceeded, *i.e.*, the different NNs make very different predictions, the corresponding geometry is assumed to lie in a conformational region with too few training points, even if the rest of the properties are predicted reliably. It is then necessary to expand the training set by computing the quantum chemistry data for this geometry. Along a dynamics run, the threshold for the error between predictions made by the NNs is adapted by multiplication with a factor of 0.95 until the conformational space is sampled sufficiently to make accurate predictions without any additional reference calculations.

An ensemble of two NNs is used not only during the initial adaptive sampling period, but also for the production dynamics simulations in order to check the accuracy of the NN predictions and to discover undersampled regions of conformational space. After 10 ps, the threshold for the RMSE between NN forecasts is not reduced anymore but kept at the previous value when a new data point is added to the training set and NNs are retrained. More details on criteria for the thresholds and iterations are discussed in the ESI.†

### 2.3 Multi-layer feed-forward NNs

For the sake of making predictions of the quantum chemical properties of molecules, multi-layer feed forward NNs are applied.^49^ For training of NNs, we use as input the matrix of inverse distances in order to achieve translational and rotational invariance in the relations established between the predicted properties and the nuclear coordinates. For prediction we use two similarly accurate NNs, with their optimal-network-architecture identified by random grid search^1^ of (hyper)parameters. Additional information on network parameters and specifications can be found in Table S1 and Section S1 in the ESI along with NN convergence during training in Fig. S1.† We assessed the quality of the used NNs by comparing them with different ML models and NNs using a different molecular descriptor on an additionally generated test set (see Section S1.3 in the ESI†). Different ML models or descriptors do not lead to a considerable improvement of the accuracy. As a different ML model we choose support vector machine for regression and linear regression as a baseline model, but our NN approaches outperform these regression models. Furthermore, the performance of our NNs is presented in Table S5† for each electronic state, separately. In this context, it is shown how the tendency towards smooth interpolation of the ML models can even correct for discontinuities present in the QC1 method (see Fig. S2†), which demonstrates the utility of our approach.

Quantum chemical properties that were learned with NNs are energies, gradients, permanent as well as transition dipole moments, and NACs. Other quantities like spin–orbit couplings can also be trained (see the analytical model in the ESI†). Although the (transition) dipole moments are not needed for the present dynamics simulation, calculating them on-the-fly enables the computation of pump–probe schemes, static-field interactions, or time-resolved spectra (see for example ref. 52 and 53). While energies are directly used for training purposes in a single NN, forces are predicted as analytical derivatives of the NNs,^54^ ensuring energy conservation.^24^,^32^,^39^ Similarly, permanent dipole moments are directly used in the training. However, couplings (as well as transition dipole moments) need to be pre-processed as they are computed from the wave functions of two different electronic states and therefore depend on the relative phases of these two wave functions. Phase inconsistencies need to be eliminated in order to avoid ill-behaved photodynamics,^55^ as is described in the following subsection.

### 2.4 Phase correction

**Fig. 2:**
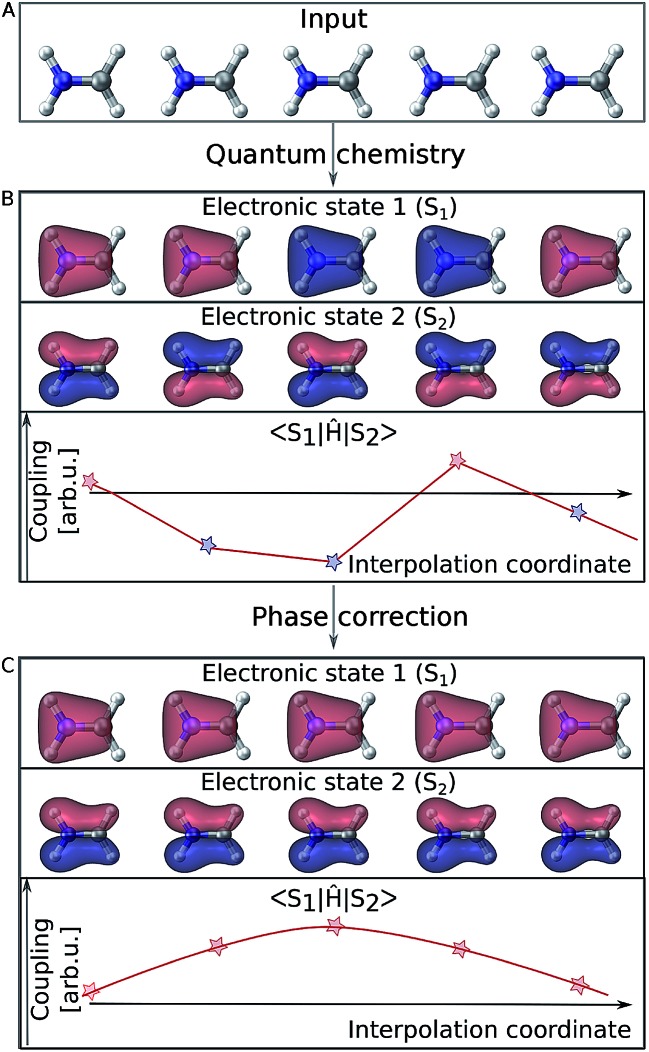
Molecular orbitals representing two different electronic states of the methylenimmonium cation, CH_2_NH_2_^+^. Panel A shows molecular geometries (with slightly different bond lengths) that are given as an input to a quantum chemistry program. The results for properties corresponding to off-diagonal matrix elements of the Hamiltonian are shown in panel B. Random signs are obtained due to random assignments of the phases of the involved wave functions. As can be seen in panel C, these random switches can be removed by phase correction, and smooth relations between a molecular geometry and any property can be found.

Electronic wave functions computed with quantum chemistry programs are usually obtained as the eigenfunctions of the electronic Hamiltonian. However, this requirement does not uniquely define an electronic wave function because multiplying it by a phase factor still returns a valid eigenfunction. Thus, in practice two wave functions computed for two very similar geometries might randomly differ in their phase factor. This problem is best visualized using molecular orbitals (see Fig. 2). For different single point calculations along an interpolation coordinate (Fig. 2A), orbitals can arbitrarily switch their sign (illustrated by their color in Fig. 2B) and so does the complete electronic wave function. As energies are obtained from diagonal elements of the general form ) and so does the complete electronic wave function. As energies are obtained from diagonal elements of the general form 〈*Ψ*_i_∣Ô∣*Ψ*_i_〉 in matrix notation, the electronic wave function enters twice and any phase is squared, thus canceling out. However, off-diagonal elements, 〈 in matrix notation, the electronic wave function enters twice and any phase is squared, thus canceling out. However, off-diagonal elements, 〉 in matrix notation, the electronic wave function enters twice and any phase is squared, thus canceling out. However, off-diagonal elements, 〈*Ψ*_i_∣Ô∣*Ψ*_j_〉, such as couplings involve the wave functions of two different electronic states and different phases do not necessarily cancel out. The example of , such as couplings involve the wave functions of two different electronic states and different phases do not necessarily cancel out. The example of Fig. 2B shows how the curves of such off-diagonal properties can be discontinuous, impeding correct learning behavior in the NN. It is thus mandatory to track the phases of all wave functions from one reference geometry to every other data point in the training set and apply a phase-correction algorithm that provides smooth curves (Fig. 2C). In this way, a virtual global phase convention is applied to all data points within the training set, with the only aim of ensuring correct NN training.

Such a global phase convention is not mathematically possible for general polyatomic molecules due to the existence of the so-called Berry (or geometric) phase.^56^ Due to the latter, the phase depends on the path between a given geometry and the reference point.^57^ Still the above phase correction is advantageous because it removes phase jumps from almost all parts of configurational space. This is critically necessary to make the data learnable. Only the non-removable phase jumps from the Berry phase remain, but occupy a small volume of configurational space. Hence, our phase correction is assumed to leave the dynamics mostly unaffected. For instance, successful surface hopping algorithms without phase tracking, such as the Zhu–Nakamura theory,^58^,^59^ exist and substantiate the validity of this approximation. In the case of the Zhu–Nakamura theory, dynamics are comparable to conventional surface-hopping molecular dynamics simulations propagated from NAC vectors.^59^–^63^ Note that the approximated phase correction for generation of the training set above cannot be circumvented by learning the absolute value of couplings since the relative sign between nonadiabatic coupling vectors of each atom in the *x*, *y* and *z* directions should be retained.

In order to make off-diagonal elements learnable for ML models, phases are tracked by computing wave function overlaps between adjacent molecular geometries.^55^,^64^,^65^ If the geometries are close enough, the overlaps will be sufficiently large and contain values close to +1 or –1, allowing a detection of phase changes. In cases where molecular geometries are too far apart, the overlap will generally be close to zero, offering no information about a phase change. In this case, we resort to interpolation between the two molecular geometries and iterative computation of wave function overlaps. In principle, the interpolation can be carried out between the new geometry and any geometry already inside the training set as long as the wavefunction of this previous geometry is stored. Storing the wavefunctions for at least a few geometries and identifying the most suitable one for interpolation *via* root mean square deviations of the geometry should be considered for larger and more flexible molecules.

Especially for large molecules, where many states lie close in energy, the so-called “intruder states” might become problematic. Such states are excluded at the reference geometry, but are included at another geometry due to an energy change, thus leading to small overlaps for the phase tracking algorithm. In such situations, different possibilities for adapting the phase correction algorithm should be considered. For instance, additional electronic states could be computed with QC. Those should not be included in the training data, but only used to continuously track the phase of all relevant states. This process then still stays affordable, since the additional states do not require a computation of gradients or couplings and do not have to be considered further. Additional details on the phase correction algorithm are given in Section S2 in the ESI.†

## 3 Computational details

The photodynamics simulations have been carried out with a development version of the program suite SHARC.^15^,^48^ Besides the newly developed modules for NN training and prediction, this development version also employs the pySHARC Python wrapper for the SHARC dynamics driver. This wrapper enables communication between the driver and the NN code without any file I/O and thus reduces the runtime of the program substantially.

The reference quantum chemical computations were carried out with COLUMBUS^66^ using the accurate multi-reference configuration interaction method including single and double excitations and a double-zeta basis set (abbreviated to MR-CISD/aug-cc-pVDZ and in the following sections labelled as QC1). For comparison, we carried out quantum chemical computations with another basis set, 6-31++G**, using the same MR-CISD method (abbreviated to QC2 in the following sections). NNs were implemented in Python using the numpy^67^ and theano^68^ packages. They were trained on energies, forces, dipole moments and nonadiabatic couplings, obtained with the QC1 method using the adaptive sampling scheme described above, resulting in about 4000 data points (mean absolute error (MAE) energies among all states: 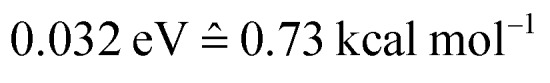; MAE forces among all states: 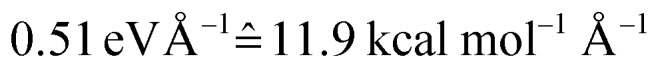; see also Tables S2, S4 and S5 in the ESI as well as Fig. S2† for analysis of different states). Using each method, QC1, QC2, and NNs trained on QC1, we simulated the dynamics of the methylenimmonium cation after excitation from the electronic ground state (S_0_) to the second excited electronic state (S_2_) over 100 fs using a time step of 0.5 fs.

Optimizations of minima were carried out with the SHARC tools that utilize an external optimizer, ORCA,^69^ where the computed energies and gradients^70^,^71^ from the NNs were fed in or those from COLUMBUS for comparison.

## 4 Results and discussion

First, a one-dimensional model was employed to test our deep learning molecular dynamics approach (see Fig. S3 in Section S3 in the ESI†). In the following, the performance of the method is demonstrated by simulating the full-dimensional photodynamics of the methylenimmonium cation, CH_2_NH_2_^+^ – the simplest member of the protonated Schiff bases. Methylenimmonium has been reported to undergo ultrafast switches between different electronic states after excitation with light.^72^ A larger member of this family is retinal, which is fundamental for vision^73^ but the methylenimmonium cation is an ideal testbed to demonstrate the applicability of NNs in photodynamics, because it is small enough to perform accurate reference photodynamics simulations for short time scales for comparison.

### 4.1 Nanosecond molecular dynamics simulation

**Fig. 3:**
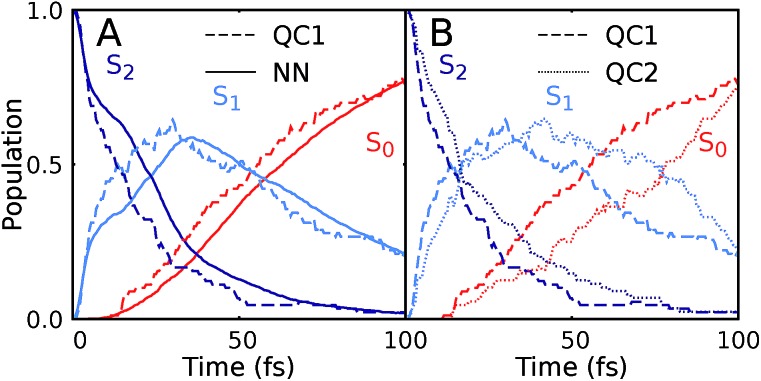
Population dynamics of CH_2_NH_2_^+^ based on deep NNs and traditional quantum chemistry: comparison between results obtained from (A) QC1 (90 trajectories) and NN (3846 trajectories) and (B) QC1 (90 trajectories) and QC2 (88 trajectories). For completeness, the populations from 90 trajectories propagated with NNs are given in Fig. S5A in the ESI along with geometrical analysis along the trajectories in Fig. S5B.†

Our NNs were trained on data obtained using the QC1 method (see details on active space in Section S4 and Fig. S4 in the ESI†). Independently with the QC1 method and with NNs, we simulated the dynamics of the methylenimmonium cation after excitation to the second excited singlet state, S_2_. As can be seen from Fig. 3A, fast population transfer from the S_2_ state to the first excited singlet state, S_1_, and back to the ground state, S_0_, takes place. The population dynamics obtained with the NN potentials and that obtained using the QC1 method agree very well. These results are also in good agreement with the literature.^72^ Both methods describe the deactivation to the ground state, S_0_, through the correct conical intersections, as will be discussed in the next subsection. A movie of one trajectory over 100 fs along with the NN potential energy curves is part of the ESI Movie S1.†

One of the first advantages of the NN driven dynamics simulations is that due to their very low computational cost, a much larger number of trajectories (3846) was simulated than what is typically possible with standard quantum chemistry (90). This enlarged statistics provides smooth population curves for the NN simulations (a comparison of the curves with an identical number of trajectories for NNs and QC1 can be found in Fig. S5A in the ESI along with analysis of energy conservation in Table S11†).

In order to estimate the magnitude of the error obtained with the NNs, we carried out a second *ab initio* molecular dynamics study with an additional, very similar, quantum chemistry method where only the double-zeta basis set is changed from aug-cc-pVDZ to 6-31++G**. As Fig. 3B shows, the differences between the two levels of theory are of the same order of magnitude as those encountered between NNs and quantum chemistry, indicating that the agreement between the methods is very good. The MAE in population between QC1 and NNs is 0.057 and between QC1 and QC2 it is 0.099. Time constants derived from dynamics with each method also agree well. The time constant from S_2_ to S_1_ is 18.3 fs according to the QC1 method, which is comparable to the QC2 method with 25.0 fs and to NN driven dynamics with 25.2 fs. The time constant obtained for transitions from S_1_ to S_0_ is 51.0 fs for the QC1 method, which is very similar to the value obtained with NNs (52.6 fs), whereas the QC2 method yields a time constant of 73.2 fs.

**Fig. 4:**
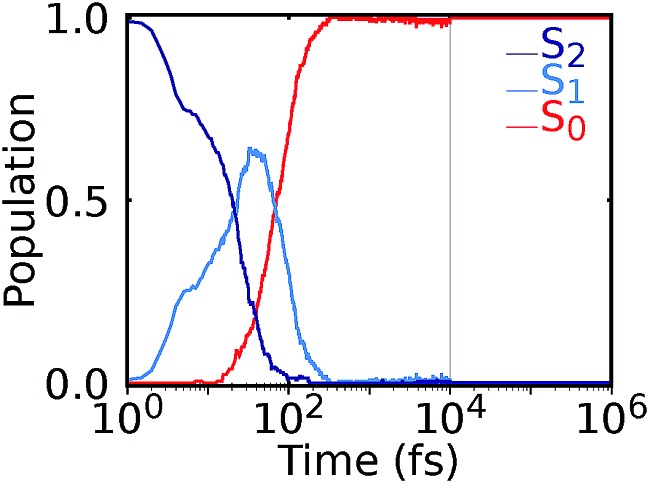
Nonadiabatic molecular dynamics simulations using deep NNs for one nanosecond. After excitation to the S_2_ state, ultrafast internal conversion to the S_1_ state takes place, followed by recovery of the S_0_ state within 300 fs. Until 10 ps, an ensemble of 200 trajectories is analyzed, followed by the population averaged from 2 trajectories.

After nonadiabatic dynamics using deep NNs has been validated for short time scales, we show the major advantage of the method, *i.e.* that it is able to overcome the problem of limited simulation time and predict long excited-state dynamics. Fig. 4 shows the population dynamics of the methylenimmonium cation on a logarithmic scale up to 1 nanosecond (ns), *i.e.*, 10^4^ times longer than they were simulated using our quantum chemical reference method. Up to 10 ps, we simulated an ensemble of 200 trajectories with 2 NNs using the adaptive sampling scheme described above in order to correctly predict events not yet learned by the NNs. After that, 2 trajectories are propagated up to 1 ns for demonstration purposes using 2 NNs. The populations are thus averaged over 200 trajectories up to 10 ps and over 2 trajectories from 10 ps up to 1 ns, respectively. As can be seen, the molecule relaxes to the ground state after around 300 fs. Due to the remaining kinetic energy a few hops between different states are recorded and can be regarded as noise. A movie of one trajectory over 10 ps is part of the ESI Movie S2.†

The propagation of a CH_2_NH_2_^+^ trajectory for 10 ps can be executed in less than 6 hours on one core, which is 300 times faster than the calculation with the quantum chemical reference method. The propagation of 1 ns took 59 days employing two deep NNs serially, whereas an estimated 19 years of computation would have been required with the quantum chemical reference.

### 4.2 Conical intersections obtained from NNs

Since NNs can provide energies, gradients, and couplings, they can also be used to optimize important points of the PES, like state minima or conical intersections. The identification of conical intersections is the target of many quantum chemical studies as they are commonly deemed as the most probable geometries for radiationless transitions between electronic states of the same spin multiplicity. Due to their special topology with discontinuous first derivatives, the surroundings of a conical intersection pose serious challenges to the NN training.^45^ As the photodynamics critically depends on a correct representation of these surroundings, here we perform some tests to validate their accuracy.

**Fig. 5:**
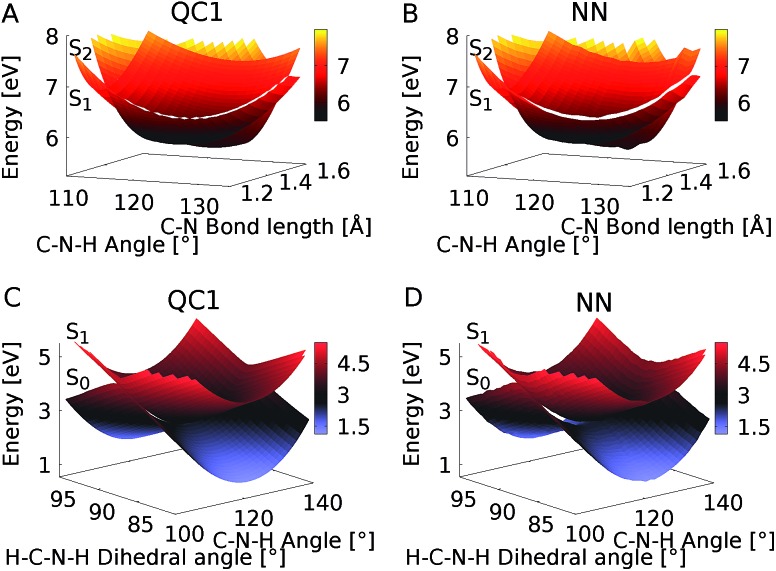
Potential energy scans around the minimum energy conical intersections obtained with QC1 of the S_2_ and S_1_ states (A and B) and S_1_ and S_0_ states (C and D). Panels A and C show the PESs calculated with QC1, while panels B and D illustrate NN potentials. See the caption of Fig. S7 in the ESI† for clarification of the dihedral angle.

To this aim we optimize two minimum energy conical intersections in CH_2_NH_2_^+^, one between the S_2_ state and the S_1_ state and another one between the S_1_ state and the S_0_ state. We use the QC1 method and NNs to perform potential energy scans around the minimum energy conical intersections optimized at the QC1 level of theory. As can be seen from Fig. 5A–D, typical curved seams of conical intersections between the S_2_ and S_1_ states (Fig. 5A (QC1) and 5B (NN)) and the S_1_ and S_0_ states (Fig. 5C (QC1) and 5D (NN)) are obtained around the minimum energy conical intersections.^74^ The NNs get the shape of this seam correct with slightly larger energy gaps between the crossing surfaces due to the fact that NN potentials need to be differentiable at any point. Analysis of 408 (for the S_1_/S_0_ CI) and 302 (for the S_2_/S_1_ CI) configurations around the minimum energy conical intersections – identified by an energy gap smaller than 0.8 eV according to the QC1 method – showed that on average, the gaps are overestimated by 0.068 eV for S_1_/S_0_ and by 0.014 eV for S_2_/S_1_ by our NNs. As can be seen from Fig. 5, the potentials around the S_1_/S_0_ CI are flatter than the potentials around the S_2_/S_1_ CI, indicating that hopping geometries are closer to the CI in the latter case and that the molecules can also hop farther from the CI in the former case.

**Fig. 6:**
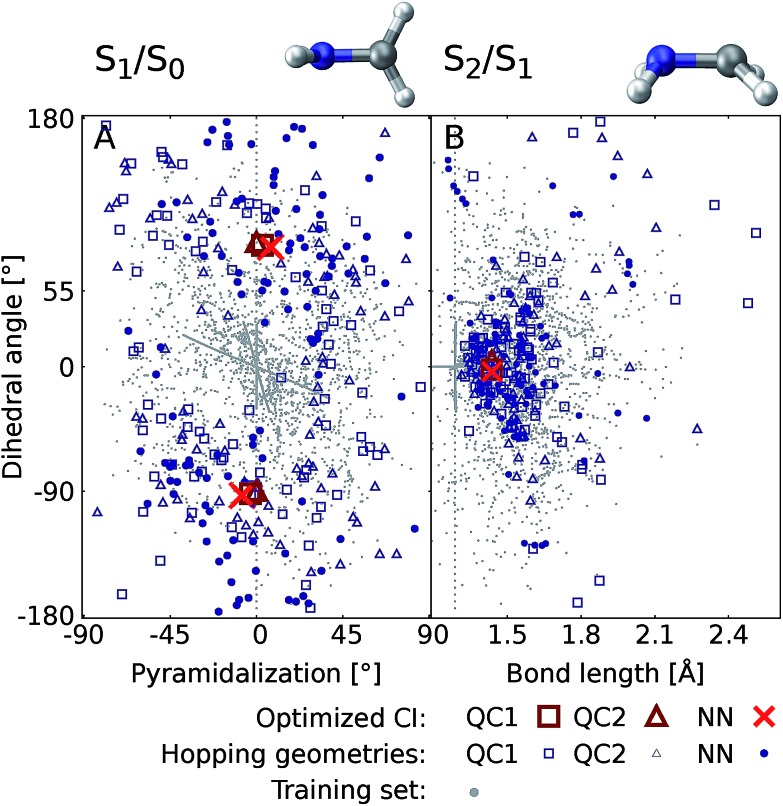
Scatter plots showing the distribution of hopping geometries obtained with QC1, QC2, and NN as well as optimized S_1_/S_0_ (A) and S_2_/S_1_ (B) minimum energy conical intersections (CIs) along with the geometries that make up the training set with 4000 data points. The actual geometry is depicted on top (geometrical parameters are given in Fig. S7B†). A zoom-in of the regions near the optimized points is shown in Fig. S7A in the ESI† together with a definition of the dihedral and pyramidalization angles.

Fig. 6 shows the scatter plots of the optimized geometries of the minimum energy conical intersections projected along two important coordinates together with the hopping geometries and the geometries contained in the training set. As can be seen, the hopping geometries between the S_2_ and S_1_ states are mainly located close to the optimized geometry of the minimum energy conical intersection, while the hopping geometries in the case of the S_1_/S_0_ crossing are more widely distributed around the optimized geometry. As a consequence, the S_2_/S_1_ crossing is sampled more comprehensively, since more trajectories pass by near the minimum energy conical intersection. This observation also explains the larger NN energy gap obtained for the second crossing, the S_1_/S_0_ CI, in Fig. 5.

The optimizations of the minimum energy conical intersections were independently performed with the trained NN, as well as with the QC1 and QC2 methods for comparison. The optimized molecular geometries (shown in Fig. S6 along with Cartesian coordinates in the ESI†) agree well. As can be seen, the driving force for the transition from the S_2_ state to the S_1_ state is an elongation of the C–N bond in combination with a bipyramidalization. The torsion of the molecule further leads to internal conversion to the ground state, S_0_. Additionally, each method results in a comparable distribution of hopping geometries around the optimized points, which in practice is of utmost importance^75^ for describing the population transfer in the simulations correctly. There are very few NN hopping geometries at either large pyramidalization angles (S_1_/S_0_ CI) or long C–N bonds (S_2_/S_1_ CI), compared to the QC trajectories. This finding correlates with the distribution of training set geometries, which are also absent in these regions of the PES (see the grey circles in Fig. 6). Configurations obtained *via* sampling of normal modes are clearly visible by a dense alignment of data points. However, the configurations obtained *via* adaptive sampling are mostly centered in the middle of the plot for the S_1_/S_0_ CI and close to the optimized CI for the S_2_/S_1_ crossing, explaining the smaller distribution of NN hopping geometries. Further analysis showed that geometries at large bond lengths are approximately 4 eV higher in energy than the geometries close to the optimized minimum energy conical intersection in the case of the S_2_/S_1_ crossing. Therefore, trajectories simulated during adaptive sampling probably did not visit those regions of the PES. In the case of the S_1_/S_0_ crossing, this effect is less pronounced and the geometries with a large pyramidalization angle are approximately 1–1.5 eV larger in energy than the configurations close to the optimized CI, indicating again the much flatter potential.

## 5 Conclusions

We demonstrate that deep NNs are able to accelerate nonadiabatic excited-state molecular dynamics simulations by orders of magnitude, thus overcoming the constraints of limited time scales and limited statistics. Our approach offers an automatic learning procedure by implementation of adaptive sampling for excited states, which opens new avenues for studying the photodynamics of complex systems on long time scales relevant for chemistry, biology, medicine, and materials design, for which the PESs cannot be explored in advance with conventional *ab initio* techniques. Offering access to the precision of high-level quantum chemistry methods at only a fraction of the original computational cost, we expect this setup to become a powerful tool in several research fields.

## Conflicts of interest

There are no conflicts of interest to declare.

## Supplementary Material

Supplementary information

Supplementary movie

Supplementary movie

Supplementary information

Supplementary information

Supplementary information
