# Supplementary material for: Machine learning enables long time scale molecular photodynamics simulations
Source: Chem Sci. 2019 Aug 5;10(35):8100–7. doi: 10.1039/c9sc01742a (PMC6849489; doi:10.1039/c9sc01742a)
Supplement: Supplementary file 6 [file SC-010-C9SC01742A-s006.pdf]

Electronic Supplementary Material (ESI) for Chemical Science.
This journal is © The Royal Society of Chemistry 2019

Electronic Supplementary Material (ESI) for Chemical Science.
This journal is © The Royal Society of Chemistry 2019

Supporting Information: Machine learning enables long time scale molecular photodynamics simulations

Julia Westermayr,[a] Michael Gastegger,[b] Maximilian F. S. J. Menger,[a,c] Sebastian Mai,[a] Leticia González[a] and Philipp Marquetand*[a]

S1 Methods

S1.1 Neural networks (NNs)

Multi-layer feed forward neural networks (NNs) have been implemented in python using the numpy[1] and theano[2] packages.

To find (hyper)parameters of the optimal-network-architecture NNs to best fit the relation between a molecular geometry and its corresponding excited-state properties, we have automated a random grid search[3] and adapted the learning rate, one of the most critical parameters during training.[4] The optimization of several parameters of NNs was done for the initial training set consisting of 100 data points for the analytical model and 992 data points for $CH_2NH_2^+$. After the adaptive sampling, the training set increased to twice its initial size. Each nuclear configuration was given to the NNs as the matrix of inverted distances. For optimal performance, we tested several aspects of the NN architecture, like the type of non-linear basis function (hyperbolic tangent and shifted softplus function, $ln(0.5e^x + 0.5)$) used, the number of neurons, the number of hidden layers, as well as other parameters, such as the learning rate, lr in the following equations, the L2 regularization rate, the number of epochs, the batch size, and a constant factor, η in equation 2, that regulates the influence of forces in the update step. Emphasis was put on optimization of the L2 regularization rate with respect to a given batch size and the learning rate.

For each quantity that has to be predicted, a separate NN was used, except for the gradients. The latter enter directly into the NNs dedicated to the potential energy prediction. The NNs for the analytical model consisted of one or two hidden layers, whereas deep NNs with 6 hidden layers were applied for training and predictions of properties of $CH_2NH_2^+$. We chose the NNs with respect to their error on the validation set as well as the form of their loss function. For the initial training set, we always sampled two different variables of the NN architectures. The learning rate was first scanned during a couple of iterations from values of 10^{-7} to 10^{0} along with the L2 regularization rate (values between 10^{-4} and 10^{-14}) using a fixed number of neurons (50) and hidden layers (4). After getting an idea about the magnitude of the L2 regularization rate, the decay factor as well as the step number for annealing the learning rate were evaluated. The decay factor was sampled from 0 to 1, whereas values between 0.9 and 1.0 seemed to give best NN training. The number of epochs, after which the learning rate should be annealed, was sampled from 1 to 1000 with values

[a] Institute of Theoretical Chemistry, Faculty of Chemistry, University of Vienna, 1090 Vienna, Austria.
[b] Machine Learning Group, Technical University of Berlin, 10587 Berlin, Germany.
[c] Dipartimento di Chimica e Chimica Industriale, University of Pisa, Via G. Moruzzi 13, 56124 Pisa, Italy.
* Corresponding author. E-mail: philipp.marquetand@univie.ac.at

between 1 and 100 leading to best results. After this, the type of basis function was analyzed. The hyperbolic tangent turned out to give fair results for nonadiabatic couplings (NACs) and dipole moments, whereas the shifted softplus function was slightly better for energies and gradients. We further tried batch sizes of 1, 2, 5, 10, 32, 64, 100, 128, 256, 500, and 992 for the initial training set (batch sizes of 2000 and 4000 were also tested for the larger and final training sets) along with the learning rate. A batch size of 500 turned out to lead to good training performance. The number of hidden layers was evaluated from 1 hidden layer up to 8 hidden layers along with the number of neurons from 10 to 100. For energies, gradients and dipole moments the number of neurons was set to 50 and for networks trained on NACs a slightly larger number of neurons was chosen. After assessing those parameters, the learning rate as well as the L2 regularization rate were sampled again. Now, a narrower space of those parameters was sampled relying upon the previously defined values. The influence of the decay factor and update step for annealing the learning rate was further reassessed. 4 Hidden layers were used for training set sizes up to around 2000 data points, afterwards 6 hidden layers slightly improved the NN accuracy.

Each time new parameters were sampled, we trained around 20-100 networks depending on the space to cover for a given parameter. The large initially sampled space of (hyper)parameters was narrowed after the initial training set was expanded and assumed to be a good starting point for random grid search. There were several sets of parameters that led to very similar performance and the two with the lowest error on a validation and training set were chosen for carrying out dynamics simulations.

S1.2 NN training

In general, training aims at fitting the weight parameters of a NN in order to minimize a cost function, which usually represents the mean-squared error (MSE) between predictions by NNs and reference data. For this task, we used Adam (adaptive moment estimation),[5] a stochastic gradient descent optimization algorithm. In combination with Adam, an exponential learning rate decay was applied.[3] Therefore, a decay-factor, f_{lr}, with values between 0 and 1, as well as a step size were defined. The step size gives the number of epochs that have to be passed after lr is adjusted according to the following equation:

$$lr = lr \cdot f_{lr} \tag{1}$$

Quantities, which can be related to a nuclear configuration and predicted by implemented NNs are energies as well as corresponding gradients, spin-orbit couplings, NACs, and dipole moments. For predictions of energies, it is favorable to include gradients in the minimization process.[6] The cost function,

$$C_{E,F} = \frac{1}{M}\sum_{m}^{M}(E_m^{NN} - E_m^{QC})^2 + \frac{\eta}{M}\sum_{m}^{M}\frac{1}{3N_m}\sum_{\alpha}^{3N_m}(F_{m\alpha}^{NN} - F_{m\alpha}^{QC})^2 \tag{2}$$

thus depends on two terms, with m running over all molecules (total number M), and α running over all Cartesian coordinates of atoms (total number 3N). The first part of Equation 2 is the MSE between energies predicted by the NN, E_m^{NN}, and reference-data energies, E_m^{QC}, obtained from quantum chemical calculations. The second part of this equation represents the MSE of molecular

forces, once derived from NNs, $F_{m_\alpha}^{NN}$, and once calculated with quantum chemistry, $F_{m_\alpha}^{QC}$. The constant η regulates the influence of forces on the overall cost function and is set to 1 here. All different electronic states were treated within one NN, thus the errors entering the cost function are for all electronic states.

The reference data set was always split into a training and a validation set in a random fashion using a ratio of 9:1. For training and prediction, inputs and outputs, X, were scaled with respect to the mean, μ, and standard deviation, σ, of the training set according to equation 3.

$$s = \frac{X - \mu}{\sigma} \tag{3}$$

An early stopping mechanism was used to control overfitting. Additionally, the convergence of the loss functions of the training and validation set were checked manually, ensuring, e.g., that their order of magnitude is similar. The training set of the analytical model consisted of 100 equidistant data points containing values of x between 0.4 and 1.8 (with x being the one degree of freedom of the harmonic oscillators, see Table S7). Two initial training sets were generated for $CH_2NH_2^+$: one via sampling along each degree of freedom as well as a torsional mode and another one by executing dynamics simulations with SHARC but starting each trajectory from the same geometry, i.e., the equilibrium geometry, with sampled velocities according to a Wigner distribution.[7] The excitation window for these simulations was set to 7-11 eV. The training set sampled along normal modes and the torsional mode yielded a better initial fit of the different computed properties, thus the focus was set only on this set for $CH_2NH_2^+$. The initial data set was insufficient for excited-state dynamics simulations and was expanded via an adaptive selection scheme as described in reference.[6] For this reason, two slightly different sets of NNs (NN1 and NN2, see Table S1 for the differing parameters and Table S2 for the mean absolute error (MAE) obtained with those NNs) were chosen to start dynamics simulations by replacing quantum chemical calculations with predictions made by NNs. The mean, $\overline{M}$, of fitted properties, $\widetilde{M_J}$, of each NN, J,

$$\overline{M} = \frac{1}{J}\sum_{j=1}^{J} \widetilde{M}_J \tag{4}$$

is given to SHARC to propagate the nuclei. Further, the root mean-squared error (RMSE) between those predictions, M_σ, was calculated on-the-fly:

$$M_\sigma = \sqrt{\frac{1}{J-1}\sum_{j}^{J}(\widetilde{M}_J - \overline{M})^2}. \tag{5}$$

Inputs according to a region of the PES that has not been visited yet could be detected on-the-fly by setting a threshold for the RMSE of each quantity predicted by NNs. The threshold is set to 18.8 kcal/mol (0.03 H) for the energies in the beginning and adapted to smaller values during sampling similar to reference[6] by multiplication with a factor of 0.95 up to 10 times. Whenever this threshold is exceeded, predictions by NNs are deemed untrustworthy and need to be recomputed

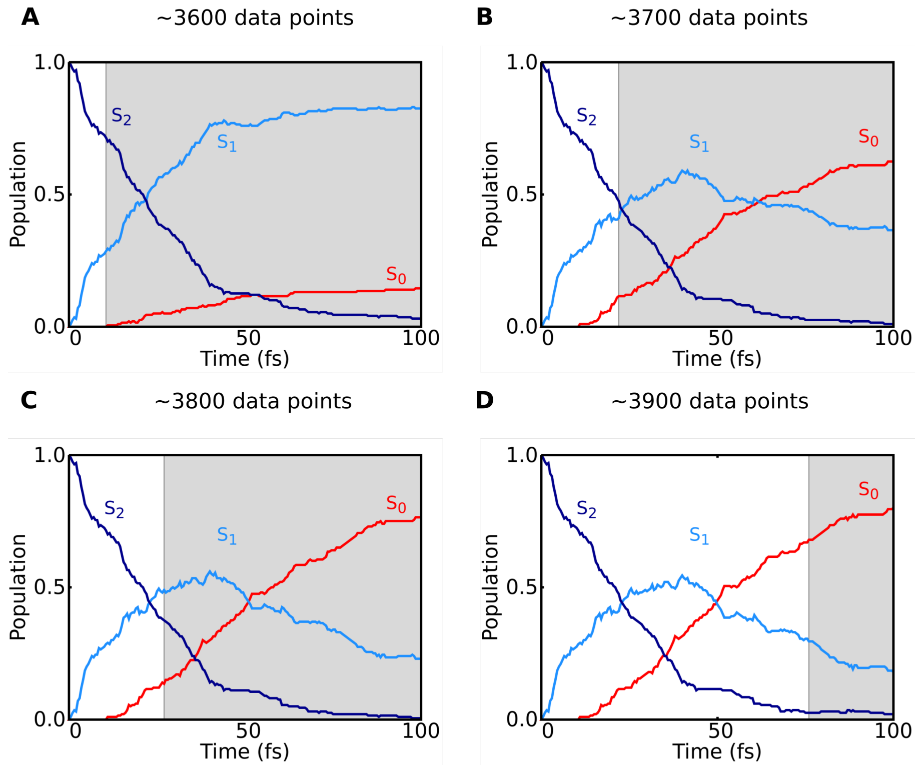

Fig. S1 Predictions of dynamics with deep NNs during the last iterations of adaptive sampling. (A) Dynamics simulations with deep NNs trained on 3600 data points. The grey area indicates the time span, where trajectories were stopped by the adaptive sampling algorithm due to running into regions with insufficient training data. (B-D) Convergence towards the correct dynamics with NNs trained on approximately 3700, 3800, and 3900 data points. See Fig. S5 for the reference dynamics. Stopping of trajectories by the sampling algorithm is shifted to later times with increasing training set size, as indicated by the grey areas.

---

with quantum chemistry as well as added to the training set. This is done for each property independently and as soon as one property is predicted unreliably, a new QC reference calculation is carried out. The NNs are retrained and previous weights are not used as a guess for training NNs. For the dipole moments, the threshold was set to 0.5 a.u. (atomic units). The latter was kept constant, since dipole moments do not directly enter into the dynamics in our current approach but are implemented for future research purposes.

For the RMSE of the NACs, the threshold is initially set to 0.25 a.u., reduced adaptively, and raised intermediately to 3.0 a.u. after a training set size of 3600 points is reached. This intermediate raise makes it possible to focus only on geometries close to conical intersections, where the NAC is verly large. These are necessary to describe the dynamics accurately. Consequently, the population dynamics converges towards the correct behavior, as can be seen in Fig. S1 for the last iterations of the sampling procedure.

As already mentioned, the predefined threshold is multiplied by a factor of 0.95 after a new conformation is detected, recalculated with QC and the NNs are retrained. The initial value was adapted by this factor after each training cycle for adaptive sampling, when the training set is still built. For the long dynamics simulations (after 10 ps), two NNs are still used for detecting undersampled regions of conformational space but threshold for the RMSE between the NNs is now kept constant (at a value of 0.05 a.u. for energies). The training set size increases only slightly (by 58 samples) during this time and are not considered to lie within the previously visited regions

Table S1 Selected parameters to construct NNs trained on data of $CH_2NH_2^+$ and carry out computations given in Figs. 3-6 of the main text and Figs. S4-6 of the ESI. NN1 describes the first NN that was defined, whereas NN2 describes the second one. The influence of the forces for training of energies was set to 1.

Property	Energy (NN1)	Dipole moments (NN1)	NACs (NN1)
Number of hidden layers	6	6	6
Number of neurons per hidden layer	50	50	74
Batch size	500	500	500
Learning rate, lr	$5.33 \cdot 10^{-3}$	$6.20 \cdot 10^{-6}$	$5.54 \cdot 10^{-5}$
Decay factor, f_{lr}	0.994	0.9999	0.994
Update steps for annealing of lr	3	1	64
L2 regularization rate	$3.32 \cdot 10^{-8}$	$8.63 \cdot 10^{-7}$	$3.99 \cdot 10^{-8}$
Basis function	Shifted softplus	Hyperbolic tangent	Hyperbolic tangent
Property	Energy (NN2)	Dipole moments (NN2)	NACs (NN2)
Number of hidden layers	6	6	6
Number of neurons per hidden layer	50	50	79
Batch size	500	500	500
Learning rate, lr	$3.49 \cdot 10^{-3}$	$2.74 \cdot 10^{-6}$	$1.22 \cdot 10^{-5}$
Decay factor, f_{lr}	0.990	0.993	0.984
Update steps for annealing of lr	5	7	79
L2 regularization rate	$5.33 \cdot 10^{-7}$	$2.39 \cdot 10^{-5}$	$2.62 \cdot 10^{-8}$
Basis function	Shifted softplus	Hyperbolic tangent	Hyperbolic tangent

Table S2 Mean absolute error (MAE) and root mean-squared error (RMSE) of predicted properties averaged over all states for the training set of the methylenimmonium cation containing 4000 data points.

	Energy [H]	Gradients [H/Bohr]	Dipole moments [a.u.]	NACs [a.u.]
MAE	0.00117	0.0189	0.0971	0.156
RMSE	0.00225	0.0395	0.208	1.13

of the PES. Those are considered to be sampled as dense as before and new data points are thus not supposed to change the performance of NNs in already passed regions of the PES. The trained model parameters used in this work can be obtained from the file NN_model_parameters.txt.

S1.3 Performance of NNs

In this subsection, we want to assess the performance of the generated NN potentials by comparing to different ML models and by an in-depth analysis of the predictions specific for each electronic state. We evaluate the accuracy by using ML models trained on the training set of the methylenimmonium cation computed with the *QC1* method (see section S4 below). We want to point out here, that the QC reference dynamics simulations are not included in the training set and thus, reproducing the dynamics can be considered as an indirect test. However, we cannot use the points from the QC reference dynamics for a direct comparison in a straightforward manner, since these points are not phase corrected. In order to evaluate the accuracy of the NN potentials, we generated an out-of-sample test set, that contains energies and forces as well as NACs (and dipole moments for completeness) of 770 data points additionally generated via sampling of linear combinations of different normal modes of $CH_2NH_2^+$. This test set is phase corrected and is made available with the training set (see file training+test_set_CH2NH2+.zip, in SHARC format). We compare our NN model with another NN model using the Coulomb matrix[8] (referred to as NN/Coulomb in the following) with 21 features (inputs) instead of the inverse distance matrix (NN/inv.D. in the following) with 15 features as a molecular descriptor. We further generate cross-products of all entries of the inverse distance matrix and use the resulting vector as descriptor (referred to as NN/poly.D. since polynomials of the inverse distances are contained). The dimension of poly.D. is thus squared compared to inv.D., hence a more accurate description and higher accuracy could be expected. To compare between different ML models, we analyze the performance of two additional regression models: Linear regression (LR) computed as a baseline model and support vector machine for regression (SVR) is used for additional comparison. In both cases, the same molecular descriptor (inv.D.) as for the reference NNs is taken.

S1.3.1 Computational details for the ML models

For training NN/Coulomb, LR and SVR models, we use the training set containing 4000 data points. We proceed in the same way as for the NN/inv.D. model described above and scale inputs and outputs for training and prediction according to equation 3. In the same manner as for the NN/inv.D. model, we sample different hyperparameters of the network architecture for the NN/Coulomb model and NN/poly.D. model (see discussion in section S1.1), but as a starting point we take optimal hyperparameters already found for the NN/inv.D. model (see Table S1). Since no better performance is obtained using different hyperparameters for the NN/Coulomb model, we keep them unchanged and only change the molecular descriptor from the matrix of inverse distances to the Coulomb matrix[8], **C**, that additionally includes the atomic charges in the representation. The diagonal elements, C_{ii}, which are absent in the inv.D. descriptor, are constant values defined as

$$C_{ii} = \frac{1}{2} Z_i^{2.4} \tag{6}$$

and off-diagonal elements, C_{ij}, are computed as

$$C_{ij} = \frac{Z_i Z_j}{\mid R_i - R_j \mid}. \tag{7}$$

Z_i in equations 6 and 7 refers to the nuclear charge of atom i and R_i is the position of atom i. All electronic states are treated within one NN. Again, forces are trained together with potential energies and predicted as their derivatives. A separate NN is used for training of NACs. In case of the NN/poly.D. model, a set of slightly different hyperparameters is chosen from hyperparameter search for energies and gradients and given in Table S3. The hyperparameters for nonadiabatic couplings are kept unchanged from NN/inv.D. models.

Table S3 Selected parameters to construct NN/poly.D. models trained on 4000 data points of $CH_2NH_2^+$. The parameter, η in equation 2, to control the influence of the forces for training of energies is set to 1. The parameters for NNs trained on NACs are depicted in Table S1.

Property	Energy
Number of hidden layers	6
Number of neurons per hidden layer	50
Batch size	500
Learning rate, lr	$3.57 \cdot 10^{-3}$
Decay factor, f_{lr}	0.950
Update steps for annealing of lr	62
L2 regularization rate	$6.10 \cdot 10^{-8}$
Basis function	Shifted softplus

To carry out LR and SVR, scikit-learn[9] is used. Each electronic state is treated separately and the forces and NACs are trained independently from the energies. Ordinary least squares LR is used as implemented in scikit-learn. For SVR, we tested different kernel types, mainly linear kernel, radial basis function and polynomials. For final training and prediction, radial basis functions are used. The penalty parameter of the error term is set to a value of 100 and the rest of the parameters are kept unchanged from default values, because the performance does not change considerably by setting different parameters.

S1.3.2 Comparison of different ML models

First, we seek to evaluate the performance of our NNs by comparison to other ML models. In our experiments, we carry out dynamics simulations with 2 NNs. Therefore, we compute the MAE averaged over all states for energies, forces, and NACs on the test set of 770 data points with two models. For the NN/inv.D. models, NN1 and NN2 are used, as they are in dynamics simulations. For the rest of the models, we split the data set of 4000 points in different training and validation sets using a ratio of 9:1 and use different initial weights. The results are given in Table S4 (for completeness, the MAE on dipole moments for our model is 0.168 a.u., but will not be discussed here). As can be seen in the table, the NNs with different descriptors reach similar accuracy. Especially, the inverse distance matrix and the Coulomb matrix perform very similarly, as would be expected from such closely related descriptors. Due to the similarity, we keep the simplest of these descriptors (inv.D.) for further analysis. SVR is worse than the NN approaches with an error

for energies and gradients approximately twice as large as for NNs. The SVR also delivers worse predictions of NACs compared to the NNs. LR, as a baseline model, has a MAE that is about a factor of 10 larger than the one of SVR for energies and gradients and about 1.5 times larger for NACs, demonstrating the utility of our NN approach described above.

Table S4 The mean absolute error (MAE) averaged over all states for energies, gradients, and NACs on a test set of 770 data points of the methylenimmonium cation for linear regression models, LR, support vector machines for regression, SVR, and NNs with the inverse distance (inv.D.), Coulomb matrix (Coulomb), and inverse distance polynomials (poly.D.) as molecular descriptors.

Model	MAE Energy [H]	MAE Gradients [H/Bohr]	MAE NACs [a.u.]
NN/inv.D.	0.00237	0.00669	0.328
NN/Coulomb	0.00238	0.00690	0.314
NN/poly.D.	0.00197	0.00617	0.335
LR/inv.D.	0.09240	0.13902	0.471
SVR/inv.D.	0.00618	0.01169	0.382

S1.3.3 State-specific analysis of the NNs

In order to clarify whether there is a bias of our ML model toward a specific state, we evaluate the errors of our reference NN/inv.D. model on energies and gradients on the same test set of 770 data points for each electronic state separately. As can be seen in Table S5, the MAE of the S_2-state energies and gradients is approximately twice as large as the one of the two lower states. Analysis of different reaction coordinates of the PES reveal problems within QC calculations that become pronounced especially in critical regions of the PES and lead to erratic potential energy curves. To show this problem, a scan along a reaction coordinate that includes two avoided potential energy curve crossings of the molecule is exemplified in Fig. S2. As can be seen, a jump in the S_2 potential energy curve is obtained with the *QC1* reference method. The source of this behaviour might be due to higher electronic states that enter along the reaction coordinate ("intruder states") or new electronic configurations of the molecule that were not included in the active space from the beginning. However, the NNs used in this work (panel A in Fig. S2) do not reproduce this discontinuity and predict smooth potential energy curves, certainly due to the use of gradients in the loss function for the training. As a consequence, the MAE obtained for the S_2 state must be artificially higher than for the rest of the states.

Table S5 The MAE on energies and gradients of each electronic state on the test set of 770 data points obtained with the NNs used in this work to carry out dynamics simulations.

State	MAE Energy [H]	MAE Gradients [H/Bohr]
S_0	0.00176	0.00444
S_1	0.00200	0.00670
S_2	0.00335	0.00893

For completeness, we carry out the same scan with the other ML models and as expected, the potential energy curves obtained with NN/Coulomb (panel B in Fig. S2) are very similar to the

ones obtained with NN/inv.D. (panel A) and NN/poly.D. (panel C). The same trend as in Table S4 is also obtained for the other models. The SVR model (panel E) gets the potential energy curves slightly worse than the NNs and LR is far off from the reference potential energy curves (panel D).

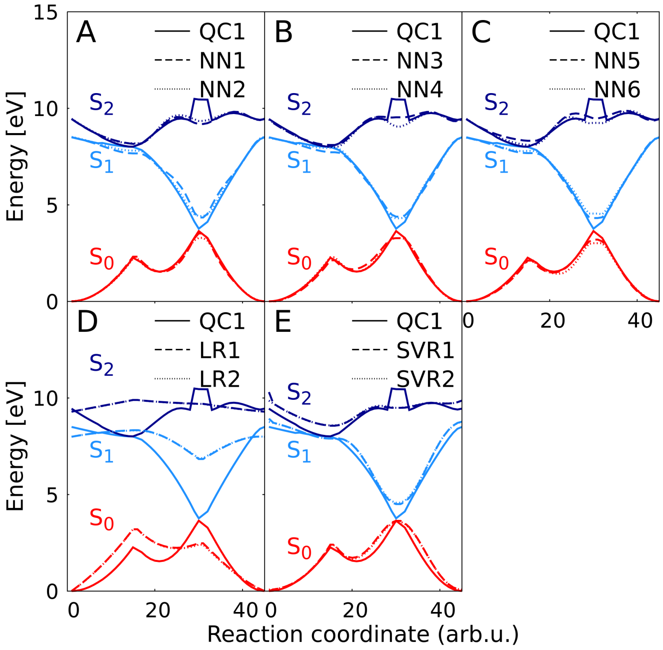

Fig. S2 Scan along a reaction coordinate of $CH_2NH_2^+$ showing two avoided crossings, one between the S_2 and S_1 state and one between the S_1 and S_0 state computed with QC1 (continuous line) and different ML models (dashed and dotted lines). (**A**) NN1 and NN2 are the reference NNs used in this work (NN/inv.D.), (**B**) NN3 to NN6 are again multi-layer feed-forward NNs, but with the Coulomb matrix[8] (NN3 and NN4: NN/Coulomb), (**C**) and polynomials of the inverse distances (NN5 and NN6: NN/poly.D.) as molecular descriptors. (**D**) LR1 and LR2 indicate linear regression models, (**E**) and SVR1 and SVR2 are support vector machines for regression. In case of the *QC1* calculation, erratic behaviour of the S_2 potential energy curve can be seen.

An important feature of the ML models used here is their tendency towards a smooth interpolation of the potential energy surfaces (see Fig. S2). As a consequence, high artificial errors and discontinuities introduced by the reference method can be compensated to a certain extent. Moreover, since all ensemble models predict the same trend in such situations (see e.g. NN1 and NN2 Fig. S2A), a low uncertainty is reported for the afflicted regions and no potentially detrimental data is added to the data set during the adaptive sampling. This feature is desired and advantageous since the ML models correct for the erratic behavior of QC1.

Nevertheless, such regions often coincide with critical regions of the PES and should be considered with care. To highlight such regions in conformational space that should be sampled more comprehensively in the training set, some other metrics could be considered. For example, very small energy gaps between different states or large coupling values are an indicator for a crossing point of two potentials. Additionally, the root-mean squared displacement (RMSD) from a new configuration to all other configurations contained in the training set could be calculated and a threshold could be set, which tells us when a new geometry is dissimilar to the current training set and should be computed with QC and added to the training set. Another possibility would be to add more data points from trajectories where the gradient is large, which is done in Ref.[10] for

example for the generation of a database. This is important, because during dynamics simulations the molecule will remain longer in regions where the forces are small, hence enlarging the training set mainly by data points with small gradients.[10] Another procedure is used for Shepard interpolation.[11] There, a statistical approach with no significant additional costs is used to determine the uncertainty of the potential energy by measuring its variance.

S2 Phase correction

We carried out a phase correction procedure to obtain a training set suitable for our ML algorithm. To this aim, a phase vector, $\mathbf{p}$, had to be derived for each molecular geometry containing values of +1 and -1 for each state. This vector was obtained by the computation of wave function overlaps between two different geometries. The overlap matrix, $\mathbf{S}$, between wave functions of following nuclear configurations, Ψ_k and Ψ_l, was computed using the WFoverlap code:[12]

$$\mathbf{S} = \langle \Psi_k \mid \Psi_l \rangle. \tag{8}$$

The overlap matrix has the dimension of $N^{states} \times N^{states}$, with N^{states} being the number of electronic states included in a calculation. When the molecular geometries are similar enough to each other, overlaps are sufficiently large to gain information whether a phase switch has occurred or not. Wave function overlaps for an electronic state that are close to +1 indicate that no phase change has taken place, whereas overlaps close to -1 point out a change in the phase of a wave function. Far from conical intersections, $\mathbf{p}$ usually corresponds to the diagonal matrix elements of $\mathbf{S}$ and also has the dimension of N^{states}. Whenever two PESs are in close proximity, off-diagonal elements of $\mathbf{S}$ can be larger than diagonal elements. In such cases, values of $\mathbf{p}$ depend on absolute values of row elements of $\mathbf{S}$. A threshold of ± 0.5 was set between overlaps of two following wave functions to ensure that the correct value of +1 or -1 was taken into account. Each entry, p_i, of $\mathbf{p}$ was then calculated according to Equation 9 with i and j running over all N^{states}.

$$p_i = sgn(max(\mid S_{ij} \mid) sgn(S_{ij})) \forall \mid S_{ij} \mid \geq 0.5; i,j = 1,2,...,N^{states} \tag{9}$$

However, many situations arise, in which computed overlaps between two geometries yield values close to 0. With these, it is not possible to decide reliably whether a phase change is present or not. Therefore, an interpolation of n steps between those two molecular geometries needed to be carried out, with n usually being in the range of 5 to 10. Interpolation was performed with respect to a molecule's Z matrix. Transformation from xyz format to a Z matrix format and vice versa was executed with OpenBabel.[13] The size of interpolation steps was sufficient when each computation yielded overlaps close to +1 or -1. The phase vector, $\mathbf{p}_n$, applicable to the last point, n, of the required interpolation was found by multiplication of all previous phase vectors, $\mathbf{p}_0$ to $\mathbf{p}_{n-1}$:

$$\mathbf{p_n} = \prod_{\beta=0}^{n-1} \mathbf{p}_\beta. \tag{10}$$

$\mathbf{p}_0$ always corresponds to the phase vector between the reference geometry, upon which the complete training set is phase corrected, and the first interpolation step. The reference geometry for global phase correction was chosen to be the equilibrium geometry.

If no row element of $\mathbf{S}$ had an absolute value above or equal to ± 0.5, the interpolation was stopped and more interpolated nuclear configurations between the equilibrium geometry and the one that needs to be included in the training set were generated. If there were still not enough

interpolated configurations to account for sufficiently large overlaps between two subsequent wave functions, the simulations were stopped. One reason for too small overlaps are "intruder states", which are excluded at the reference geometry, but are included at another geometry due to an energy drop. Thus, a previously included state is excluded such that the overall molecular electronic wave function changes considerably.

The phase correction of each matrix **M** – in our case, the Hamiltonian matrix, which contains energies and couplings, and the dipole matrices for each direction of the coordinate system – was carried out according to Equation 11. Matrices containing all relevant nonadiabatic coupling vectors to form the nonadiabatic coupling tensor with the dimension $N^{states}xN^{states}xN^{atoms}x3$ (with N^{atoms} being the number of atoms) were corrected according to Equation 12. Each two dimensional nonadiabatic coupling vector, $\mathbf{NAC}^{ij}$, accounts for NACs between two states i and j. It was corrected by multiplication of every element with the entry of **p** for each state i, p_i, and j, p_j.

$$\mathbf{M} = M_{ij} \cdot p_i \cdot p_j; i, j = 1, 2, ..., N^{states} \tag{11}$$

$$\mathbf{NAC}^{ij} = \mathbf{NAC}^{ij} \cdot p_i \cdot p_j \tag{12}$$

S3 Analytical Model

In order to check the performance of our deep learning molecular dynamics approach we constructed an one-dimensional, diabatic analytical model consisting of five harmonic oscillators, with the analytical interface of SHARC.[14,15] The potential energy curves are defined in Table S6. Nonadiabatic potential couplings (in contrast to nonadiabatic derivative couplings) are responsible for transitions between the ^1X and ^1A state while spin-orbit couplings coupled states of different spin multiplicity, i.e. ^1X and ^3B as well as ^1A and ^3B. These couplings as well as dipole moments between different states are described by constant values given in Table S7 and S8, respectively. A total number of 2000 initial starting points were sampled according to a Wigner distribution,[14] from which 100 were selected, excited in a range of 0-2 eV and propagated for 100 fs with a time step of 0.05 fs for the nuclear motion and 0.001 fs for the analytical solution of electronic amplitudes.

The five harmonic oscillators of the analytical model are given in Fig. S3 and represent the ground state ^1X (dark blue line in Fig. S3A), one excited singlet state ^1A (light blue line in Fig. S3A), and three degenerate excited states to mimic a triplet state, ^3B (red line in Fig. S3A). The states are coupled by both nonadiabatic potential couplings and spin-orbit couplings. Sampling of configurations along the degree of freedom leads to a training set of 100 data points and the potential energy curves can be fitted nearly exactly by NNs (solid lines in Fig. S3A). Excited-state dynamics are started from 100 different initial conditions generated in the ^1X state, each excited to the ^1A state. The time evolution of the different states using the original analytical potentials and the trained NNs (Fig. S3B) show that the predictions are comparable and each state is populated similarly after 100 fs. The MAE in the predictions of the energies is 0.00031 eV (0.01 kcal/mol) and of the gradients is 0.0024 eV/Å (0.057 kcal/mol/Å). Hyper parameters chosen for the NNs can be obtained from Table S9. This first proof-of-concept thus shows the ability of our ML method to describe excited-state dynamics including nonadiabatic potential and spin-orbit couplings.

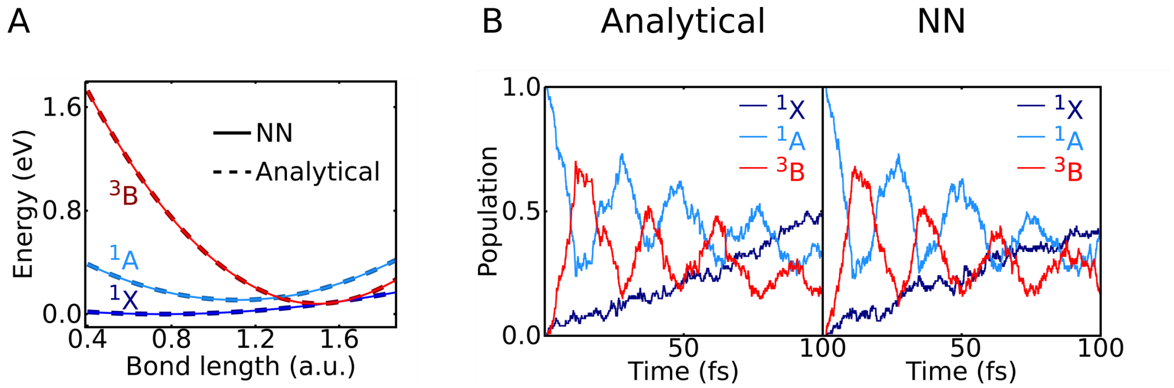

Fig. S3 Analytical model. (A) Analytical (dashed lines) and neural network (NN, solid lines) potential energy curves along an one-dimensional model. **(B)** Time evolution of the different electronic state population after excitation from the ^1X state to the ^1A state using the exact analytical setup and NNs.

Table S6 Parameters of harmonic potentials according to the equation $V(x) = 0.5k(x - x_0)^2 + E_0$.

Energetic state	k[a.u.]	x_0 [a.u.]	E_0 [a.u.]
^1X	0.01	0.756	0.0
^1A	0.04	1.115	0.004
^3B	0.1	1.50	0.003

Table S7 Couplings between different electronic states of the analytical model.

Couplings between states	^1X [a.u.]	^1A [a.u.]	$^3B(+)$ [a.u.]	$^3B(0)$ [a.u.]	$^3B(-)$ [a.u.]
^1X		0.00025	0.005-0.0001i	-0.0025i	0.005+0.0001i
^1A	0.00025		0.001+0.0025i	+0.0025i	0.001-0.0025i
$^3B(+)$	0.005+0.0001i	0.001-0.0025i			
$^3B(0)$	0.0025i	-0.0025 i			
$^3B(-)$	0.005-0.0001i	0.001+0.0025i			

Table S8 Dipole moments between different electronic states of the analytical model.

Dipole moment between states	^1X [a.u.]	^1A [a.u.]	$^3B(+)$ [a.u.]	$^3B(0)$ [a.u.]	$^3B(-)$ [a.u.]
^1X	-1.90	-0.3			
^1A	-0.3	-1.2			
$^3B(+)$			-0.3		
$^3B(0)$				-0.3	
$^3B(-)$					-0.3

Table S9 Selected parameters to construct NNs trained on data of the analytical model to carry out dynamics simulations given in Fig. S3. NN1 describes the first NN that was defined, whereas NN2 describes the second one. The influence of the forces for training of energies was set to 1.

Property	Energy (NN1)	Couplings (NN1)	Dipole moments (NN1)
Number of hidden layers	1	2	2
Number of neurons per hidden layer	13	32	36
Batch size	50	50	50
Learning rate, lr	$4.83 \cdot 10^{-3}$	$9.37 \cdot 10^{-4}$	$9.41 \cdot 10^{-4}$
Decay factor, f_{lr}	-	-	-
Update steps for annealing of lr	-	-	-
L2 regularization rate	$6.07 \cdot 10^{-10}$	$2.35 \cdot 10^{-12}$	$6.23 \cdot 10^{-10}$
Basis function	Shifted softplus function	Hyperbolic tangent	Hyperbolic tangent

Property	Energy (NN2)	Couplings (NN2)	Dipole moments (NN2)
Number of hidden layers	1	2	2
Number of neurons per hidden layer	39	32	24
Batch size	50	50	50
Learning rate, lr	$7.89 \cdot 10^{-3}$	$9.31 \cdot 10^{-4}$	$9.48 \cdot 10^{-4}$
Decay factor, f_{lr}	-	-	-
Update steps for annealing of lr	-	-	-
L2 regularization rate	$6.23 \cdot 10^{-10}$	$1.94 \cdot 10^{-12}$	$1.50 \cdot 10^{-14}$
Basis function	Shifted softplus function	Hyperbolic tangent	Hyperbolic tangent t

S4 Methylenimmonium cation

S4.1 Computational details for QC

The quantum chemical reference calculations of the methylenimmonium cation, $CH_2NH_2^+$, were done with the program COLUMBUS[16] which uses the implementation of nonadiabatic coupling vectors via Dalton[17] integrals. $CH_2NH_2^+$ possesses 16 electrons. Molecular orbitals were optimized with the state-averaged complete active space self-consistent field (SA-CASSCF) method, averaged over 3 states. The CAS was set to 6 active electrons in 4 active orbitals. The two lowest molecular orbitals representing the 1s orbitals of the C and N atom (Fig. S4A) were frozen at the MRCI level of theory. The next three lowest energetic orbitals were set to be inactive (Fig. S4B) and 4 orbitals were assigned to the reference space with 6 active electrons (Fig. S4C). Single and double excitations from the (6,4) reference space to 73 virtual orbitals yield a total of about 660000 configuration state functions. Determinants from inactive orbitals to virtual orbitals for single and double excitation are included within MRCI-SD. The frequency calculation was carried out with COLUMBUS. We performed scans with 100 points along each normal mode (Table S10) as well as with 72 points along the torsion around the central double bond in order to generate the initial training set. From these scans, we removed data points from computations that did not show proper convergence. After adaptive sampling, we ended up with a training set of 4000 data points.

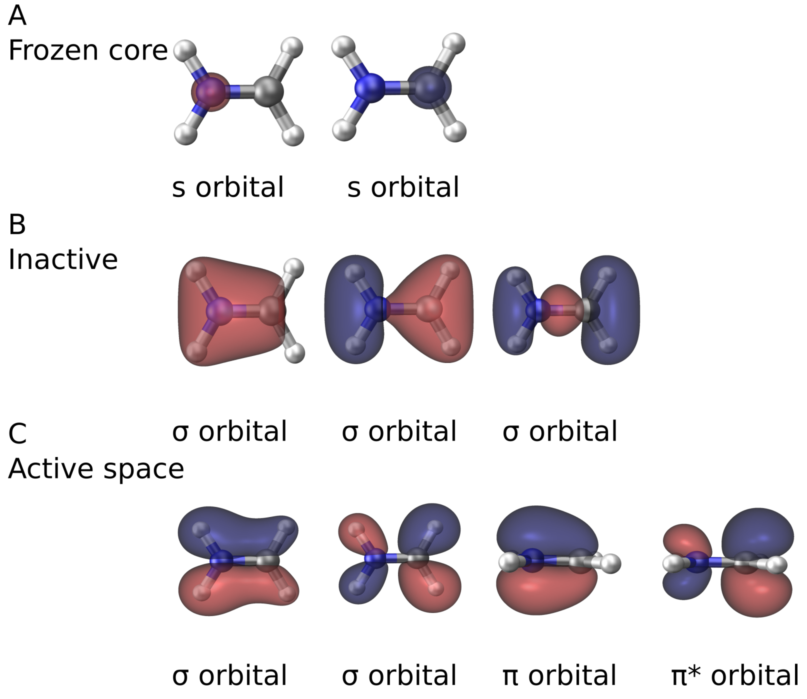

Fig. S4 Isosurface plots of the orbitals of the methylenimmonium cation. (A) Orbitals representing the frozen core. **(B)** Orbitals representing inactive doubly occupied space energetically between the frozen core and the active space. **(C)** The active space consisting of 6 electrons in 4 orbitals.

S4.2 Surface-hopping molecular dynamics

As a prerequisite for the dynamics, 1000 initial conditions were sampled from the Wigner distribution of the quantum harmonic oscillator defined by the above-mentioned frequency calculation. From these 1000 possible starting points, 200 were excited – according to the oscillator strengths in the excitation window of 9.44 ± 0.15 eV – to the brightest state, which is the second excited

Table S10 Frequencies of each normal mode of $CH_2NH_2^+$ computed with MR-CISD/aug-cc-pVDZ.

Degree of freedom (normal mode)	Frequency $[cm^{-1}]$
1	940.09
2	969.35
3	1074.45
4	1174.32
5	1368.83
6	1473.26
7	1612.35
8	1789.73
9	3223.36
10	3357.28
11	3550.29
12	3663.38

singlet state. This excitation shows mostly $\pi\pi^*$ character. Each trajectory was propagated for 100 fs with a time step of 0.5 fs for nuclear motion and 0.02 fs for integration of the time-dependent Schrödinger equation. Trajectories showing problems with energy conservation due to improper convergence of the quantum chemistry were excluded, resulting in 90 trajectories for *QC1* and 88 trajectories for *QC2*. However, the trend of the populations when all trajectories were taken into account is the same. Therefore, no bias was introduced by sorting out trajectories due to bad energy convergence within quantum chemical calculations, which appeared mainly around conical intersections due to convergence problems. Such problems do not occur in the NNs, which demonstrates another advantage of the ML approach. Fig. S5A depicts a direct comparison of the molecular dynamics computed with *QC1* (MR-CISD/aug-cc-pVDZ) and NNs. In both cases, the populations of 90 trajectories starting from the second excited singlet state are shown. Two hundred initial conditions were excited with 9.44 ± 0.15 eV and 90 trajectories reached 100 fs in the case of *QC1*. Therefore, from NN-simulations only the first 90 trajectories were used for comparison. As can be seen, the populations of each state are in good agreement when comparing both methods – as they are in Fig. 3 in the main text. In the case of the populations shown in Fig. 4 in the main text, we propagated 200 trajectories up to 10 ps. Afterwards, for demonstrating the possibility of long time scale simulations, we propagated 2 trajectories up to 1 ns. The populations up to 10 ps are averaged over 200 trajectories, the populations from 10 ps on up to 1 ns are averaged over 2 trajectories, respectively. In case of the nanosecond time scale simulations, every 100th time step, equivalent to every 50 fs, is written out by the program pySHARC. The kinetic constants discussed in the main text are obtained using the tools of SHARC.[14,18] By solving differential equations that describe the kinetics of the underlying model, a fit for the population transfer can be obtained and rate constants can be derived.

In addition to the population dynamics, we also checked whether visited molecular geometries are comparable along the trajectories. We first calculated the mean of each nuclear configuration

over time from the NNs leading to populations given in Fig. S5 and computed the RMSD to the mean molecular geometry at the respective time step of the ensemble predicted with MR-CISD/aug-cc-pVDZ, i.e. *QC1* (Fig. S5B continuous line). An analogous comparison is also carried out for *QC1* and *QC2* (MR-CISD/6-31++G**, Fig. S5B, dotted line). For completeness, we also computed the RMSD between NN and *QC2* (Fig. S5B, dashed line). All RMSDs are of comparable size, further validating our NN approach.

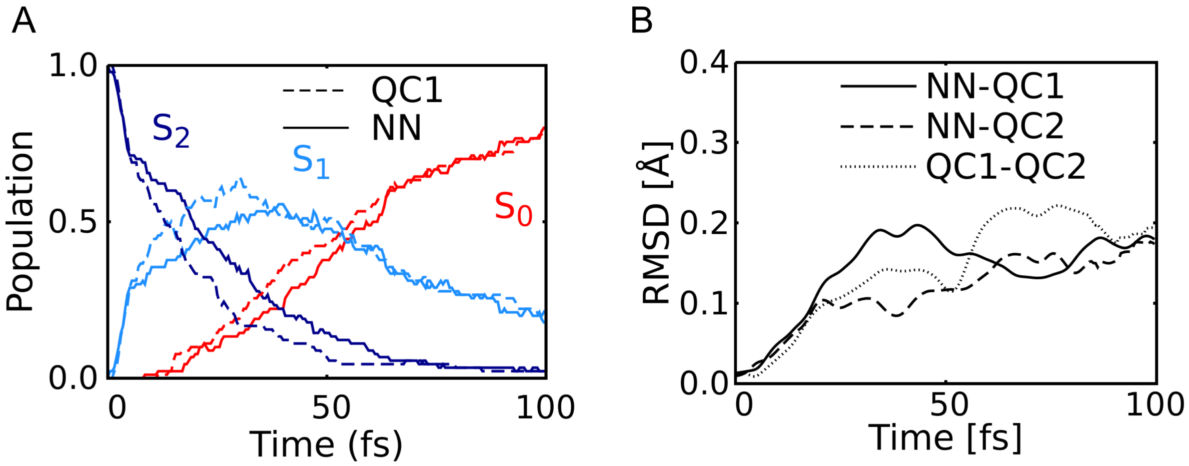

Fig. S5 (A) Comparison of dynamics from *QC1* and NN with 90 trajectories, respectively. The continuous lines show the populations of each excited singlet state calculated with a deep neural network (NN), whereas the dashed line shows the corresponding population computed with *QC1* (MR-CISD/aug-cc-pVDZ). **(B)** RMSD of the mean of nuclear configurations of each trajectory over time. NN-*QC1* is the RMSD between deep NNs and the reference quantum chemical method *QC1* (MR-CISD/aug-cc-pVDZ), NN-*QC2* is the RMSD between deep NNs and the *QC2* method (MR-CISD/6-31++G**), whereas *QC1*-*QC2* is the RMSD between *QC1* and *QC2*.

Further, we checked the energy conservation of trajectories with the reference method *QC1* and NNs independently. For both methods, we computed the mean and standard deviation (Std.) of the total energy along a trajectory, where we once allow hopping and once force the trajectories to stay in the S_2 state after excitation. Results are shown in Table S11. As can be obtained, the mean total energy as well as the Std. along trajectories propagated for 100 fs are comparable among the methods. However, the Std. increases slightly when hops are forbidden between different energetic states. This trend is obtained with both methods. Moreover, when a time step of 0.05 fs is used instead of 0.5 fs for classical propagation of the nuclei, the energy conservation improves, which meets expectations. Worth mentioning is that, in the case of *QC1*, trajectories which show large steps in total energy or do not reach 100 fs at all are removed prior to analysis. This is not the case for NNs, as all trajectories were suitable for analysis and no prior selection was carried out.

S4.3 Conical intersections (CIs)

With each method, *QC1*, *QC2*, and NNs, we optimized the geometries of the two S_1/S_0 and S_2/S_1 CIs. Optimizations were executed with the SHARC tools that utilize an external optimizer of ORCA,[19] where the computed energies and gradients[20,21] from COLUMBUS or NNs are fed in. Geometries are shown in Fig. S6. They were optimized starting from the different hopping geometries obtained with the afore-mentioned methods, as indicated in the scatter plot of Fig. 6 in

Table S11 MAE and standard deviation (Std.) of the total energy obtained with the *QC1* (MR-CISD/aug-cc-pVDZ) method and neural networks (NN). In the case of a time step of 0.5 fs for classical propagation of the nuclei, 90 trajectories were analyzed, whereas 50 trajectories were used for analysis of trajectories with a time step of 0.05 fs.

Method	Time step [fs]	MAE [eV]	Std. [eV]	Hops allowed?
QC1	0.5	10.63	0.047	Yes
QC1	0.5	10.77	0.059	No
QC1	0.05	10.73	0.011	No
NN	0.5	10.72	0.052	Yes
NN	0.5	10.73	0.061	No
NN	0.05	10.80	0.017	No

the main text and the zoom in Fig. S7. The optimized molecular geometries agree well. Cartesian coordinates are given in Table S12-S15 and are further added as ascii-files to the supplementary material.

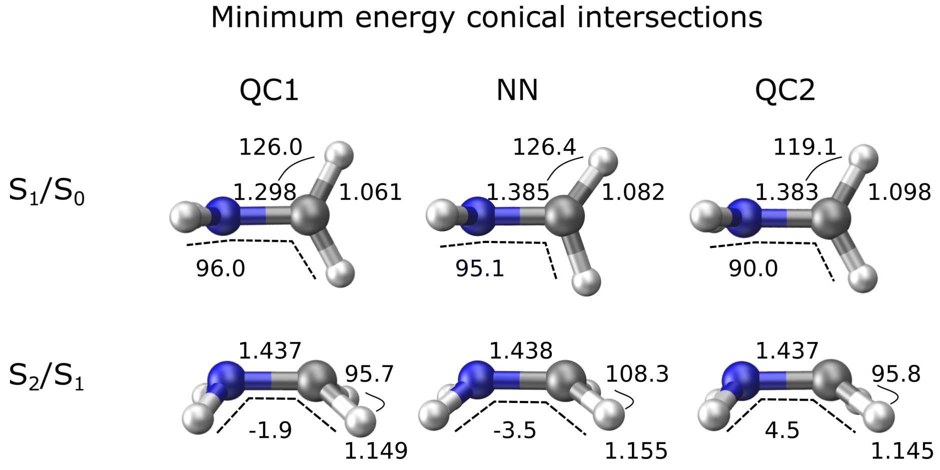

Fig. S6 Conical intersections (CIs). For each method, we could find two different CIs. *QC1* (MR-CISD/aug-cc-pVDZ) is used as the reference method to train neural networks (NNs) and compared to CIs obtained with *QC2* (MR-CISD/6-31++G**). For the two CIs, the bond length between the nitrogen (blue) and the carbon (grey) atom is shown, as well as the bond length between the carbon and one hydrogen atom. Values are given in angstrom. A dihedral angle between four atoms marked with the dashed line is given, as well as an angle between the carbon and a hydrogen atom (S_1/S_0 CI) and between two hydrogen atoms (S_2/S_1 CI).

The S_1/S_0 CI shows a rotation of the molecule around the H3-N-C-H4 dihedral angle (see Fig. S7 for the definition) of $\sim 90°$. Due to symmetry of the molecule, the CI is obtained at $\sim 90°$ as well as at $\sim -90°$. The optimized geometries agree well between all methods (*QC1*, *QC2*, NN). All methods yield hopping geometries that are distributed in a rather large region around these points. The S_2/S_1 CI, is defined by a slight bi-pyramidalization and a bond elongation between the carbon and the nitrogen atom of around 1.44 Å (see Fig. S6) compared to a bond length of around 1.29 Å at the equilibrium geometry (Fig. S7B). Again, molecular geometries obtained with different methods are very similar. The scatter plot in the right panel of Fig. S7A shows the good agreement among different methods and also depicts the distribution of data points in the final training set.

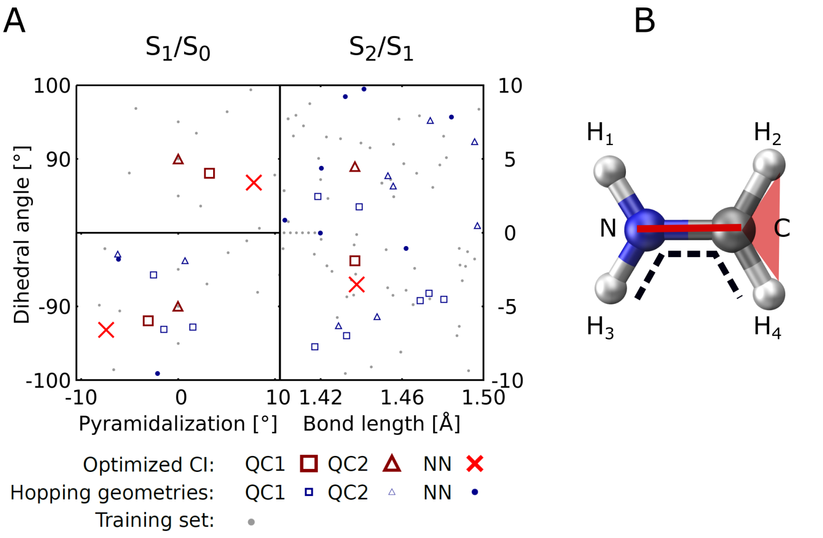

Fig. S7 Conical intersection analysis in addition to Fig. 5 in the main text. (A) Scatter plots showing the distribution of hopping geometries of each method as well as optimized nuclear configurations close to the minimum energy CI present between the S_1 state and the ground state, S_0, (left) as well as between the S_2 state and the S_1 state (right). Geometries from the training set with 4000 data points are plotted as blue circles.

(B) Shown is the equilibrium geometry of methylenimmonium cation in order to specify the atoms that describe the used dihedral and pyramidalization angle in Fig. 5 and Fig. 6 in the main text and Fig. S6 here. The pyramidalization angle that is used to describe the S_1/S_0 CI is defined by the angle between the bond between the nitrogen (N) and the carbon (C) atom (red line) and the plane that is spanned by the C atom and the two hydrogen atoms, H2 and H4 (red triangle). The angle between the planes spanned by atoms H3-N-C and N-C-H4 is chosen to distinguish geometries describing both CIs is defined by hydrogen atoms, H3 and H4, as well as by the C and N atom (dashed black line).

S4.4 Movie S1

A trajectory molecular dynamics simulation of the methylenimmonium cation, $CH_2NH_2^+$, during 100 fs after its excitation to the second excited singlet state predicted with deep NNs in combination with SHARC can be found in the supplementary material along with the potentials, on which the molecule moves at each time step.

S4.5 Movie S2

A trajectory molecular dynamics simulation of the methylenimmonium cation, $CH_2NH_2^+$, during 10 ps after its excitation to the second excited singlet state predicted with deep NNs in combination with SHARC can be found in the supplementary material.

Table S12 Cartesian coordinates of the S_1/S_0 CI obtained from deep NNs. Also available from the file *CI_opt_geoms.xyz* of the supplementary material.

Atom	x-coordinate [Å]	y-coordinate [Å]	z-coordinate [Å]
C	-0.09510	0.0547	-0.1529
N	0.0928	0.0520	1.2196
H	0.6760	0.7307	-0.6251
H	0.7899	-0.6147	1.8341
H	-0.5741	0.8014	1.7538
H	-0.7677	-0.5838	-0.7111

Table S13 Cartesian coordinates of the S_1/S_0 CI obtained from MR-CISD/aug-cc-pVDZ. Also available from the file *CI_opt_geoms.xyz* of the supplementary material.

Atom	x-coordinate [Å]	y-coordinate [Å]	z-coordinate [Å]
C	-0.067495	-0.271367	-0.083550
N	0.002414	-0.191463	1.316026
H	-0.264937	-1.250001	-0.530777
H	0.882335	-0.347469	1.825166
H	-0.826010	0.016867	1.888644
H	0.133036	0.637597	-0.658113

Table S14 Cartesian coordinates of the S_2/S_1 CI obtained from deep NNs. Also available from the file *CI_opt_geoms.xyz* of the supplementary material.

Atom	x-coordinate [Å]	y-coordinate [Å]	z-coordinate [Å]
C	0.092590	0.05507	-0.03636
N	-0.02183	0.01101	1.3961
H	0.9094	-0.2682	-0.6655
H	0.6959	-0.4079	1.9997
H	-0.7125	-0.46625	1.9366
H	-0.8898	-0.3630	-0.4760

Table S15 Cartesian coordinates of the S_2/S_1 CI obtained from MR-CISD/aug-cc-pVDZ. Also available from the file *CI_opt_geoms.xyz* of the supplementary material.

Atom	x-coordinate [Å]	y-coordinate [Å]	z-coordinate [Å]
C	0.049953	-0.345208	-0.016365
N	0.033847	-0.254299	1.417459
H	0.910037	0.088100	-0.642754
H	0.869788	0.200682	1.810525
H	-0.809643	0.202872	1.791428
H	-0.793958	0.090807	-0.662521

S5 Future perspectives

As an outlook we expect our approach to offer new possibilities to study also excited-state dynamics of larger molecules on longer timescales. By using the adaptive sampling procedure in combination with NN dynamics simulations, an initial training set can be automatically expanded until the conformational space of a molecule important for dynamics is sampled comprehensively. In the following, a short discussion on expected scaling of the methods and possibilities to treat larger molecules is given.

As a good starting point for the initial training set we suggest sampling of normal modes and linear combinations of different normal modes. Alternatively, the points obtained from a geometry optimization of critical points like CIs might deliver an even better initial set. As a guideline, we suggest from our limited experience that approximately 1000 data points should be considered for the initial training set. With this procedure, some critical regions of the potential energy surface close to the equilibrium geometry can already be covered in the training set. Further sampling of reaction coordinates that are considered to be important during excited-state molecular dynamics simulations seems to be a good guess. As an example, a reaction coordinate that involves the dissociation of an atom, can be included. Dependent on the size and complexity of the molecule as well as on the degree of initial sampling, the trajectories will be interrupted more frequently in the initial stages of an adaptive sampling run. In terms of training set sizes in general, we do not expect a linear scaling along with the number of atoms. The number of necessary data points does not only depend on the number of atoms of a molecule, but also on its excited state dynamics dictated for example by its flexibility or the excitation energy. The more processes that can take place after excitation of a molecule, the larger the space is that needs to be covered. Further, the number of states included in dynamics simulations as well as the quantum chemical reference method play a role and should be considered. In general, no rule can be given in terms of optimal training set size, but the convergence behavior of the adaptive sampling runs can serve as an indicator on whether additional reference data is required. Finally, we want to point out that using the adaptive sampling scheme – as soon as enough data points for a machine learning model can be obtained within a reasonable time frame – seems to be most efficient for sampling the relevant space visited during a dynamics simulation. We further recommend to take special care when selecting the number of electronic states. As already discussed in the main text within the section on the phase correction algorithm, it is important to compute more electronic states for molecules that have a lot of electronic states lying close in energy. However, those additional states should only be used for phase correction and computation of wavefunction overlaps and do not need to be considered for couplings or computation of forces. With this approach, problems arising due to "intruder states" during the phase correction procedure can be avoided. Regarding the network architecture, we recommend to adapt it to the complexity of the molecule and expect approximately linear scaling of both, the size of the network and its computational performance, with the number of atoms of a molecule.

Last but not least, we want to comment on non-molecule specific excited-state potentials, similar to the idea of universal force fields that are in part already available for the ground state.

We expect this to be a very complex task that has to be considered with a lot of care. If molecules show similar excited state dynamics – for example the methylenimmonium cation and the ethylene molecule[22] are isoelectronic and show a rotation along the dihedral angle for the S_1/S_0 transition – a non-molecule specific method can be thought of to describe this transition. However, the order of higher-lying states is different in these two isoelectronic species and higher excitation energies will induce completely different dynamics in the respective systems. Therefore and also due to the problematic generalization and molecule-specific couplings and excited-state potentials, we do not expect a non-molecule specific model to be available in the near future.

References

1 S. van der Walt, S. C. Colbert and G. Varoquaux, *Comput. Sci. Eng.*, 2011, **13**, 22–30.

2 Theano Development Team, *arXiv*, 2016, 1605.02688 [cs.SC].

3 I. Goodfellow, Y. Bengio and A. Courville, *Deep Learning*, MIT Press, 2016.

4 X. Glorot and Y. Bengio, Proceedings of the Thirteenth International Conference on Artificial Intelligence and Statistics, Chia Laguna Resort, Sardinia, Italy, 2010, pp. 249–256.

5 D. P. Kingma and J. Ba, ICLR 2015, 2014.

6 M. Gastegger, J. Behler and P. Marquetand, *Chem. Sci.*, 2017, **8**, 6924–6935.

7 E. Wigner, *Phys. Rev.*, 1932, **40**, 749–750.

8 M. Rupp, A. Tkatchenko, K.-R. Müller and O. A. von Lilienfeld, *Phys. Rev. Lett.*, 2012, **108**, 058301.

9 F. Pedregosa, G. Varoquaux, A. Gramfort, V. Michel, B. Thirion, O. Grisel, M. Blondel, P. Prettenhofer, R. Weiss, V. Dubourg, J. Vanderplas, A. Passos, D. Cournapeau, M. Brucher, M. Perrot and E. Duchesnay, *Journal of Machine Learning Research*, 2011, **12**, 2825–2830.

10 A. Pukrittayakamee, M. Malshe, M. Hagan, L. M. Raff, R. Narulkar, S. Bukkapatnum and R. Komanduri, *J. Chem. Phys.*, 2009, **130**, 134101.

11 K. C. Thompson and M. A. Collins, *J. Chem. Soc., Faraday Trans.*, 1997, **93**, 871–878.

12 F. Plasser, M. Ruckenbauer, S. Mai, M. Oppel, P. Marquetand and L. González, *J. Chem. Theory Comput.*, 2016, **12**, 1207.

13 N. M. O'Boyle, M. Banck, C. A. James, C. Morley, T. Vandermeersch and G. R. Hutchison, *J. Cheminf.*, 2011, **3**, 33.

14 S. Mai, P. Marquetand and L. González, *WIREs Comput. Mol. Sci.*, 2018, **8**, e1370.

15 S. Mai, M. Richter, M. Ruckenbauer, M. Oppel, P. Marquetand and L. González, *SHARC2.0: Surface Hopping Including Arbitrary Couplings – Program Package for Non-Adiabatic Dynamics*, sharc-md.org, 2018.

16 H. Lischka, R. Shepard, R. M. Pitzer, I. Shavitt, M. Dallos, T. Müller, P. G. Szalay, M. Seth, G. S. Kedziora, S. Yabushita and Z. Zhang, *Phys. Chem. Chem. Phys.*, 2001, **3**, 664–673.

17 T. Helgaker, H. J. A. Jensen, J. O. P. Jørgensen, K. Ruud, H. Ågren, T. Andersen, K. L. Bak, V. Bakken, O. Christiansen, P. Dahle, E. K. Dalskov, T. Enevoldsen, H. Heiberg, H. Hettema, D. Jonsson, S. Kirpekar, R. Kobayashi, H. Koch, K. V. Mikkelsen, P. Norman, M. J. Packer, T. Saue, P. R. Taylor and O. Vahtras, *DALTON, An Ab Initio Electronic Structure Program, Release 1.0*, 1997.

18 S. Mai, M. Richter, M. Ruckenbauer, M. Oppel, P. Marquetand and L. González, *SHARC: Surface Hopping Including Arbitrary Couplings – Program Package for Non-Adiabatic Dynamics*, sharc-md.org, 2014.

19 F. Neese, *WIREs Comput. Mol. Sci.*, 2012, **2**, 73–78.

20 B. G. Levine, J. D. Coe and T. J. Martinez, *J. Phys. Chem. B*, 2007, **112**, 405–413.

21 M. J. Bearpark, M. A. Robb and H. B. Schlegel, *Chem. Phys. Lett.*, 1994, **223**, 269.

22 B. Sellner, M. Barbatti, T. Müller, W. Domcke and H. Lischka, *Molecular Physics*, 2013, **111**,

2439–2450.
